# Optical Sensing of Microbial Life on Surfaces

**DOI:** 10.1128/AEM.03001-15

**Published:** 2016-02-19

**Authors:** M. Fischer, G. J. Triggs, T. F. Krauss

**Affiliations:** Department of Physics, University of York, York, United Kingdom; University of Bayreuth

## Abstract

The label-free detection of microbial cells attached to a surface is an active field of research. The field is driven by the need to understand and control the growth of biofilms in a number of applications, including basic research in natural environments, industrial facilities, and clinical devices, to name a few. Despite significant progress in the ability to monitor the growth of biofilms and related living cells, the sensitivity and selectivity of such sensors are still a challenge. We believe that among the many different technologies available for monitoring biofilm growth, optical techniques are the most promising, as they afford direct imaging and offer high sensitivity and specificity. Furthermore, as each technique offers different insights into the biofilm growth mechanism, our analysis allows us to provide an overview of the biological processes at play. In addition, we use a set of key parameters to compare state-of-the-art techniques in the field, including a critical assessment of each method, to identify the most promising types of sensors. We highlight the challenges that need to be overcome to improve the characteristics of current biofilm sensor technologies and indicate where further developments are required. In addition, we provide guidelines for selecting a suitable sensor for detecting microbial cells on a surface.

## INTRODUCTION

Biofilms are ubiquitous in aqueous environments. Their formation on submerged surfaces is readily initiated by the attachment of proteins, followed by individual bacteria, which then trigger other species to colonize. In the Baltic Sea or Atlantic Ocean, for example, mature natural biofilms consist of bacteria, fungi, diatoms, protozoans, larvae, and algal spores embedded in an extracellular polymeric substance (EPS) matrix (1–3). Most of the microbial activity in aquatic environments is found at the solid-liquid or air-liquid interface (4). Biofilms play an important role in the aquatic food chain and the biogeochemical pathways of carbon, nitrogen, hydrogen, sulfur, and phosphorus, and they are the major component of the earth's biodiversity; a comprehensive review can be found in reference 5. In general, the hydrodynamics of the liquid phase, environmental conditions, such as temperature or pH, the physiological and metabolic state of cells, and substrate conditions have been shown to affect levels of bacterial adhesion. Commonly, the properties of the surface, such as charge, hydrophobicity, topography, and the identities of the exposed chemical groups, interact with physicochemical properties of microbial cells, influence cell attachment, and therefore may vary under different environmental conditions. An overview summarizing recent studies on bacterium-surface interactions is provided by Tuson and Weibel (6). Biofilms also form on industrial aquatic installations, initiating processes such as corrosion and biofouling (7). Microbial colonization can also reduce heat or mass transfer on heat exchangers, condensers, and membranes (8). In a medical context, bacterial biofilms play a significant role in our daily lives, as they cause major problems in dental hygiene, infectious diseases, and infections related to medical implants. They may develop on all types of clinical devices, such as heart valves, contact lenses, and urinary, endotracheal, intravenous, and other types of catheters (9, 10). Mature biofilms can tolerate antimicrobial substances in concentrations 10 to 1,000 times (11) higher than those used for planktonic microorganisms and are even more resistant to phagocytosis. The National Institutes of Health of the United States estimated that more than 80% of the bacterial infections in the human population are biofilm related and that patient mortality associated with biofilms is substantial (12). In particular, device-related infections constitute a major cause of bacterial infections in hospitalized patients (13–15). The detection of microbial cells is an active field of research, and several monitoring techniques based on electrical conductivity or electrical capacity (16), calorimetry (17), and friction and pressure drop (18), as well as sound (19) and electromagnetic radiation, have been developed. All biofilm monitoring techniques can be divided into (i) direct measurements relating to the mass or the cell density and (ii) indirect measurements of metabolic activity and products such as liquids or gases. Several overviews of sensor strategies are available (20–27). Here, we focus on optical sensors that are label free and that directly detect the optical properties of the film via the optical density or via spectroscopic signatures. Moreover, we present examples for the application of optical biofilm sensors in the field.

**FIG 1:**
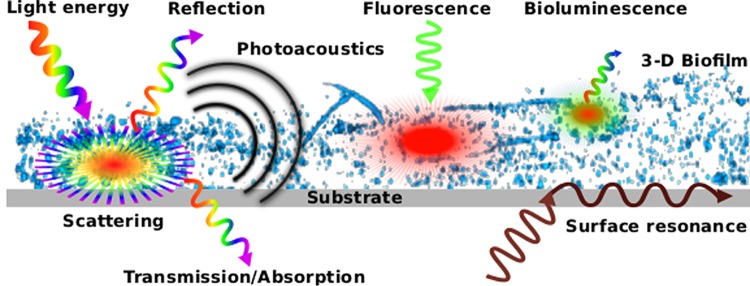
Overview of the main interactions between light and biofilms that are utilized for exploring microbial surface colonization.

To allow for a comparison of different types of biofilm sensors, we define several parameters that help delineate the various types. First of all, sensors must be able to discriminate between organic material simply suspended in water and that attached to a surface; ideally, the sensor should also have sufficient depth resolution in order to account for the biofilm's three-dimensional (3-D) structure. Second, the sensor should have a sufficiently large substrate surface and the capability to probe several square millimeters in order to average across the typically inhomogeneous settlement characteristics of biofilms, which form highly patchy clusters of cells several hundred micrometers in diameter (28, 29). Third, a wide dynamic range is required to quantify the full range from initially adsorbed bacterial cells up to complex biofilm communities. Finally, autonomous operation with a sufficiently long operational time and rapid and easy signal acquisition are likewise desirable criteria. An increasing number of biofilm monitoring systems are based on the detection of light across the entire spectrum, i.e., from UV to visible light, and on infrared (IR) radiation. The corresponding interaction between optical radiation and biofilm matter can then be utilized to examine biofilms and their formation dynamics: scattering, absorption/transmission, reflection, photoacoustics, fluorescence, bioluminescence, and surface resonance. These interactions are summarized and schematically shown in Fig. 1.

## TURBIDITY MONITORING OF BIOFILMS

A number of different methods have been introduced to assess the optical density of biofilms and to provide information about the film's thickness. Measuring the optical turbidity or the loss of intensity by light scattering is typically performed in a wavelength range of 600 to 1,300 nm to minimize absorption. This wavelength window is also known as the “therapeutic window,” as it maximizes the penetration depth into tissue and biofilms. For example, a monitoring system based on turbidity, which can detect biofilm accumulation and removal with a near-infrared (NIR) source (950-nm wavelength), was introduced by Tinham and Bott (30). The NIR emitter and detector were mounted on the outside of glass tubes on which the biofilm was grown, and the resulting signal was correlated with the thickness of the biofilm. This method could detect film thicknesses from 30 μm to 250 μm and was used to study the impact of flow velocities and pH on biofilm growth. Similarly, Meyer et al. (31) and Ghodssi et al. (32) developed an arrayed microfluidic platform that utilized optical density to monitor the formation of Escherichia coli biofilms. They used a setup consisting of off-the-shelf components, such as a 660-nm LED array and two external photodiodes, to measure the transmitted light. This setup enabled them to detect changes in optical density of 0.06%, which corresponds to a detection limit of 6 μm for biofilm thickness. While both of these sensors exhibit a large dynamic range in terms of biofilm thickness, they operate in transmission, which makes it impossible to distinguish between attached and free-floating particles and microorganisms.

## SURFACE-SENSITIVE SENSORS

To achieve a higher sensitivity, the challenge is to detect the light signal at the surface while avoiding interference by the bulk water, thereby increasing the signal-to-noise ratio; in this context, the signal-to-noise ratio is also referred to as the biofilm collection efficiency (33). To meet this challenge, the total internal reflection is used, as it provides surface sensitivity on a low background, and only the evanescent field of the reflected light interacts with the biofilm. As an example of this approach, Zibaii et al. used a tapered fiber to detect a bacterial biofilm (E. coli) with a minimum cell density of 6 × 10^3^ cells/cm^2^ (34). The penetration depth of this sensor, which operates at 1,558 nm, is calculated to be ∼0.42 μm.

The advantage of this fiber optic sensor is the intrinsic background suppression through the utilization of the evanescent field, which is highlighted by the low detection limit that can be achieved compared to that of the transmission-type sensors (30–32); transmission-type sensors achieve a detection limit of only ∼10^7^ cells/cm^2^. Another type of surface wave, namely, a surface plasmon, can be used for the same purpose. Surface plasmons have similar penetration depths, which are on the order of 180 nm and are demonstrated by Kee et al. (35). They used surface plasmons to detect bacterial (E. coli) attachment and growth. The plasmonic sensor operates on the principle of detecting the shift in the extraordinary optical transmission peak caused by a change in the surrounding refractive index. The sensor surface was functionalized with E. coli-specific antibody to particularly capture E. coli cells, and the bacterial growth was monitored. The bacteria covered the sensor surface (0.01 mm^2^) with an average density of 2.2 × 10^3^ to 1.7 × 10^5^ cells/cm^2^. These sensors show an excellent sensitivity for bacterial cells, but the sensing area and penetration depth remain small, which make them unsuitable for detecting complex and patchy biofilms in the field. To improve the penetration depth, a reverse-symmetry waveguide structure with an imprinted sinusoidal surface-relief grating (36) can be used, where the substrate refractive index is lower than the cover medium and the evanescent field can be extended to penetration depths of 278 and 592 nm for the transverse electric (TE) and the transverse magnetic (TM) modes, respectively. This reverse symmetry waveguide sensor with an integrated grating coupler has been used to detect the accumulation of E. coli cells on the sensor surface (8 mm^2^) and achieved a minimum detection limit of 6 × 10^3^ cells/cm^2^ (37).

To probe a larger fraction of a cell, a modified sensor with a penetration depth of 727 nm was used to detect normal human dermal fibroblast (NHDF) cells (38–40). In addition, another group of waveguide sensors, called metal-clad waveguide sensors, employs a metal layer to increase the probing depth of the waveguide modes (41). The sensor performances were tested with NHDF cells and human keratinocyte HaCaT cells, which lead to a detection limit of 8 × 10^2^ cells/cm^2^ (42). Compared to that of bacterial biofilm sensors, the detection limit of the previously described sensor is 3.6 × 10^5^ bacterial cells/cm^2^, assuming that an E. coli cell covers an area of 1 μm^2^ (0.5 μm in width by 2 μm in length) and that the measured HaCaT cell (24 μm in diameter) covers an area of 452 μm^2^.

**FIG 2:**
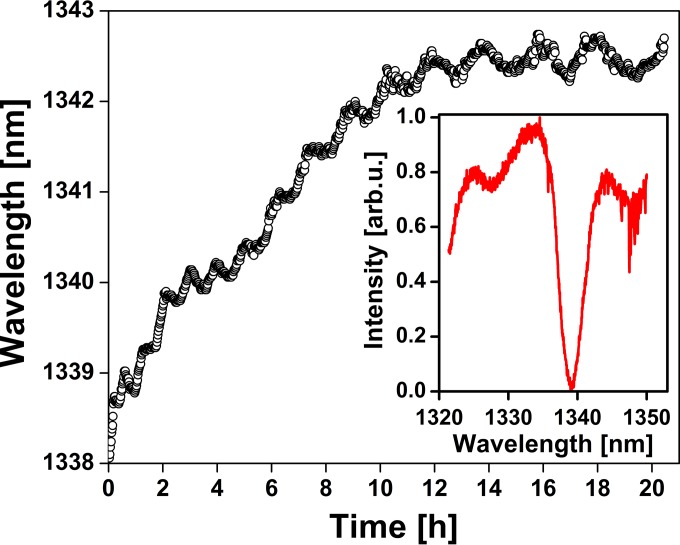
Resonance shift of a 2-D photonic crystal caused by microbial surface colonization. The bacterial density after the experimental period was 3 × 10^4^ bacterial cells/cm^2^, which corresponds to a microbial surface coverage below 0.25%. The inset shows the resonance dip that is being tracked at a wavelength of around 1,340 nm. arb.u., arbitrary units.

We recently explored microbial settlement on a 2-D photonic crystal fabricated in silicon on a transparent and flexible substrate. Interaction of the biofilm with the surface induces a change in the refractive index at the interface and causes a shift in the resonance wavelength, which is proportional to the mass of the biofilm bound to the surface. The design parameters for the 220-nm-thick silicon slab used here were a square array (3 mm^2^) with a periodic hole structure of period *a* equal to 600 nm and radius *r* equal to 120 nm. We fabricated the biofilm sensor by a procedure similar to the procedure described by Scullion et al. (43) and in combination with a polydimethylsiloxane (PDMS) microfluidic channel integrated with the photonic structure. We measured the biofilm establishment within the first hours, as shown in Fig. 2.

Due to their intrinsic wavelength scalability and small footprint, sensors based on photonic crystals hold great potential for the miniaturization and the integration of the major device components, such as a light source and detector, into a single chip. A summary of the aforementioned sensors is given in Table 1.

**TABLE 1:** Summary of surface sensitive biofilm sensors

Sensor type (reference)	Surface material (area)	Penetration depth (nm)	Detection range (cells/cm^2^)
Tapered optical fiber (34)	SiO_2_ (0.17 μm^2^)	420	6 × 10^3^ to 6 × 10^7^
Plasmonic nanohole arrays (35)	Au (0.01 mm^2^)	180	2 × 10^3^ to 2 × 10^5^
Photonic crystal resonance	Si (3 mm^2^)	189	3 × 10^3^ to 1.2 × 10^7^^*a*^
Grating-coupled planar optical waveguide (37)	Polystyrene (8 mm^2^)	592	6 × 10^3^ to 1.2 × 10^6^

a Calculated detection range.

## SPECTROSCOPIC BIOFILM STUDIES

Bioluminescence occurs spontaneously in a biofilm when, for instance, luciferin is oxidized in the presence of ATP and the enzyme luciferase (44). Bacterial bioluminescence has been used to quantify the impact of biofilm saturation on porous media (45, 46); the emitted bioluminescence signal of Pseudomonas fluorescens correlated directly with sessile bacteria counts in the range of 10^5^ to 10^7^ cells/cm^2^ (47). Pathogen-host interactions during biofilm infection with Candida albicans in a live host were monitored *in situ* and noninvasively by dynamic imaging and by quantification of the growth phase-dependent bioluminescence intensity (48–50). As bioluminescence is limited to only a few organisms, this sensor strategy is not applicable to most naturally occurring biofilms.

In contrast, microorganisms typically exhibit fluorescence upon excitation in UV. This fluorescence is due to the presence of, e.g., tryptophan, which is an essential amino acid that is present in all living organisms with a maximum absorption at a wavelength (λ_ex_) of ∼280 nm and a peak emission (λ_em_) of ∼350 nm. Therefore, tryptophan can be used as an indicator of biofilm growth and as an indicator for the presence of biomass in general. Furthermore, the fluorescence provides quantitative information, as the signal strength directly correlates with the bacterial cell number. For example, detection minima of 5 × 10^6^ cells/cm^2^ (51), 5 × 10^5^ cells/cm^2^ (52), and, more recently, 4 × 10^3^ cells/cm^2^ (33) have already been achieved.

Complementarily to fluorescence methods, absorption spectroscopy can be used in the mid-IR range, where biomass exhibits typical absorption spectra in the 4,000- to 600-cm^−1^ wavenumber range or in the 2.5- to 16-μm wavelength range (53). The IR absorption bands correspond to the presence of proteins, lipids, polysaccharides, polyphosphate groups, and other carbohydrate functional groups within the biofilm. A particularly promising and surface-sensitive variant of the technique is attenuated total reflectance Fourier transform infrared (ATR-FTIR) spectroscopy, where the penetration depth of the evanescent field is ≤1 μm. For example, the absorbed IR radiation of the C=O and C—N stretching vibration bands corresponding to the amide I (1,647 cm^−1^ or 6.07 μm) and amide II (1,548 cm^−1^ or 6.46 μm) groups (54–58), respectively, was used to follow the development of the biofilm, with a limit of detection of 5 × 10^5^ cells/cm^2^ (23). In another example, ATR-FTIR spectra (1,800 to 750 cm^−1^ or 5.6 to 13.3 μm) of bacteria (Pseudomonas putida) with a resolution of 4 cm^−1^ growing on a hematite-coated germanium crystal showed a shift to higher frequencies (∼15 cm^−1^) in the carboxylate signal (∼1,400 cm^−1^ or 7.1 μm) than in those of the samples obtained from free-floating cells (59). These results indicate that carboxylate groups from macromolecules on the cell wall of the bacteria can structurally couple to the surface. There is a corresponding small shift (5 cm^−1^) in the spectrum of polysaccharides, which are biopolymers of microbial origin in which biofilm microorganisms are embedded (60), in the region of 1,200 to 950 cm^−1^ (8.3 to 10.5 μm) whenever bacteria are attached or unattached to the surface; this indicates their involvement in the cell attachment process.

Raman spectroscopy is yet another spectroscopic technique that affords fingerprinting of specific molecules. Raman spectroscopy is based on the effect of inelastic light scattering and provides the vibrational spectra of biological samples. Since all biologically relevant molecules, such as proteins, nucleic acids, carbohydrates, and lipids, exhibit distinct spectroscopic signatures, the combination of spectral features seen in a Raman spectrum provides a unique fingerprint of a given molecule. Typically, a near-IR wavelength monochromatic laser source is chosen in order to prevent thermal effects on samples and to avoid the background fluorescence that is commonly observed in biological materials. As a result, Raman microscopy made it possible to identify ammonium-oxidizing bacteria in a biofilm cultured in synthetic wastewater by the identification of the unambiguous stretching vibration of ammonium, hydrazine, and hydroxylamine as shown by Pätzold et al. (61). The authors used a 100-μm fiber in combination with an objective, which delivers the light of a 532-nm laser source that has a penetration depth of up to 70 μm into the biofilm and a detection volume of 1 μm^3^. This means that the Raman signal is very localized. Similarly, it has been possible to distinguish between different strains of the species Staphylococcus epidermidis (62) as well as between the diverse species of the Legionella genus and the strains of Pseudomonas aeruginosa, Klebsiella pneumoniae, and E. coli (63), which can either form biofilms or remain free-floating. Moreover, it is possible to distinguish between the two species Streptococcus sanguinis and Streptococcus mutans within a mixed biofilm with 97% accuracy (64, 65). The main issue with Raman spectroscopy is that it has a very low efficiency, i.e., Raman signals are typically 10^6^ times weaker than fluorescence signals, and it requires sophisticated filters and detection systems.

To increase the Raman signal, metal nanoparticles that cause hot spots and concentrate the light field can be used. This technique is referred to as surface-enhanced Raman spectroscopy (SERS) (66), and the Raman signal scales 10^4^-fold with the field increase, such that enhancements up to 10^11^ are possible (67). In the context of biofilms, SERS can be used to characterize the chemical composition of the biofilm matrix by the evolution of the relevant Raman peaks, as shown by Chao and Zhang (68). The authors used three model bacteria, E. coli, Pseudomonas putida, and Bacillus subtilis, to cultivate biofilms together with silver nanoparticles. As the biofilm grew, the relative contents of carbohydrates, proteins, and nucleic acids in the biofilm matrix increased significantly. Remarkably, the authors were able to see that the content of lipids increased only in the Gram-negative biofilms (E. coli and P. putida), not in the Gram-positive biofilm (B. subtilis). The results also indicated that polysaccharides increased significantly from initial bacterial adhesion to the formation of the mature biofilm, which has also been shown with ATR-FTIR spectroscopy (59). In another example, SERS was used to monitor the development of a dual-species biofilm formed by two model bacteria, Brevundimonas diminuta and Staphylococcus aureus, on a mixed cellulose ester membrane surface (69). The analysis of the Raman spectra allowed the authors to characterize dynamic changes in the dominant species of the biofilm with culture time and provide chemical information. It was shown that S. aureus cells attached rapidly to the membrane surface and dominated the dual-species biofilm during the first hours but then detached and were outcompeted by B. diminuta cells. These examples demonstrate clearly that Raman spectroscopy enables real-time differentiation of bacteria on the level of the individual species. It also enables the study of the specific chemical compounds involved in the attachment of bacteria as well as their identification within a cell down to the single-molecule level.

An alternative spectroscopic technique is the combination of light absorption and sound detection, the so-called pulsed photoacoustic spectroscopy (PPS) technique. The technique is based on the absorption of pulsed electromagnetic radiation inside of a biofilm and its conversion into heat. Due to the corresponding thermal expansion of the biofilm caused by the light pulse, a pressure wave that can be detected by microphones or piezoelectric transducers is generated. The intensity of the detected sound signal is proportional to the optical absorption coefficient of the biofilm, which in turn can be correlated to the thickness of the film (70). Depth-resolved measurements with a resolution of 10 μm were performed at three different wavelengths; at a wavelength of 440 nm, the incident light is absorbed by pigments, while at 1,580 nm and at 2,240 nm, there are absorption bands of water and carbohydrates, respectively (71, 72). A time-resolved measurement, which records the time delay between the light pulse and the arrival of the pressure wave, then allows for a depth-resolved investigation of the biofilm. In comparison with optical coherence tomography (OCT), PPS provides a chemical composition with inferior spatial resolution, while in comparison to FTIR, PPS provides information about the chemical composition as a function of depth.

The information made available by each of the methods listed above, together with their respective benefits and drawbacks, is outlined in Table 2.

**TABLE 2:** Summary of spectroscopic methods for the analysis of biofilms

Spectroscopic method (reference[s])	Information provided	Strengths	Weakness(es)
Bioluminescence (45–50)	Cell density and coverage, ATP concn	Low background, high signal-to-noise ratio, no photobleaching, inexpensive instrumentation	Limited to a few organisms harboring the *lux* gene
Fluorescence (33, 51, 52, 101)	Cell density, coverage area, microbial activity, DNA/protein concn	Specific intrinsic fluorescence, low exposure times, low detection limit	Limited chemical information, broad spectral features
FTIR-ATR (23, 54, 55, 59)	Cell density, presence of proteins, lipids, polysaccharides, and functional groups	Sensitive to heteronuclear functional groups, surface sensitive, high spectral resolution	High water absorption, broad spectral features
Raman (61, 62, 64, 65)	Differentiation between bacterial strains by specific functional groups	Sensitive to homonuclear molecular bonds, occurs at all wavelengths, high spatial resolution, suited for aqueous solutions	Weak signal, background fluorescence, long exposure time, complex instrumentation
SERS (68, 69)	Differentiation between bacterial strains by specific functional groups, single-molecule detection	Very high enhancement of Raman scattering, high spectral specificity	Variation of the local field enhancement, addition of metal nanoparticles, background fluorescence
PPS (70–72)	Biofilm thickness, pigments, and carbohydrates, specific functional groups	Wide dynamic range, no background drift, high penetration depth	Complex instrumentation, low depth resolution

In summary, the majority of the sensors focus on the differences in the chemical compositions of the biofilms or on the identification of a single species within a biofilm, but generally they do not provide exact quantitative concentration ranges of the monitored biomolecules (except for fluorescence, which can be quantitative). In this respect, analytical techniques are complementary to sensing techniques, and they provide more information on the very nature of the biofilm, its growth mechanism, and its evolution.

## BIOFILM IMAGING

Light microscopy enables the imaging of microorganisms down to the level of a single cell. For the calibration of a biofilm sensor, microscopy combined with computerized image analysis is therefore an important tool. Clearly, microscopy techniques can also be used for investigating the qualification and quantification of biofilm growth, which is the subject of a recent review (74, 85).

Hyperspectral chemical imaging combined with confocal microscopy is an up-and-coming research field with great potential for the discovery of new insights about biofilm formation encompassing spatially resolved spectral data obtained through a variety of modalities (e.g., Raman microscopy [75–78] and FTIR microscopy [79–81] chemical imaging). It goes beyond the capabilities of conventional imaging and spectroscopy by obtaining spatially resolved spectra from objects at spatial resolutions down to the level of a single cell (82). In addition, Bhartia et al. demonstrated that deep-UV (DUV)-laser-induced native fluorescence can image a single bacterial cell with a DUV microscope with a resolution of 300 nm over an area of 700 μm^2^ (83). The excitation at wavelengths between 200 and 250 nm results in intrinsic fluorescence response signatures associated with bacterial cells or spores and peaks in a UV range between 270 and 400 nm. This response is derived from the combinatorial absorption and fluorescence signatures of intrinsic proteins, free amino acids, nucleic acids, flavins, and other aromatic compounds that have been concentrated in the cells.

**FIG 3:**
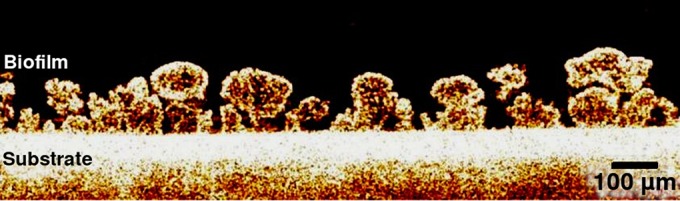
*In situ* OCT image of mushroom-like biofilm structures in a drinking water system. Reprinted with permission from Elsevier (87).

A related imaging technique that additionally provides depth resolution is optical coherence tomography (OCT). OCT is an interferometric method that typically employs near-infrared light and is also used as an investigative method, rather than being used solely to sense biofilm growth. In OCT, the backscattered signal depicts the relative optical density distribution in the biofilm, with a typical depth resolution of 5 to 10 μm, which is provided by the coherence length of the source. The ability of OCT to monitor transient processes occurring in microbial films was highlighted by demonstrating a temporal resolution on a time scale of seconds to minutes (73, 84). OCT enables the visualization of the complete heterotrophic biofilm structure, including the substrate, pores, and connected structures simultaneously (86, 87). Recently, Dreszer et al. (87) demonstrated the suitability of OCT for *in situ* measurements of biofilm thickness and morphology during biofilm development, biofilm detachment, and permeate flux change on a rectangular area of 10 mm^2^ with a physical penetration depth of 1.1 mm, an axial resolution of 5.8 μm, and a lateral resolution of 8 μm. The specific advantage of OCT compared to microscopy techniques is that staining of the sample is not necessary to monitor biofilm structure, and as shown in Fig. 3, relatively large areas of 1,680 by 500 μm can be observed.

In a medical application, Nguyen et al. demonstrated the ability to noninvasively image the occurrence of a middle-ear otitis media infection that was caused by biofilms using a hand-held, high-resolution-depth-ranging OCT system (88). The superluminescent diode operating at a center wavelength of 830 nm and a bandwidth of 70 nm used in this OCT system allowed for axial resolutions of up to 4.5 μm and penetration depths of up to 2 mm. Based on the scattering properties of the tympanic membrane, a thickness of 95 μm was measured without any infection, while with infection, a 200-μm-thick biofilm was detected behind the membrane. As a downside, OCT is inherently incapable of revealing chemical information about biofilm constituents, and the spatial resolution does not allow single-cell imaging.

## SURFACE-ENHANCED IMAGING

**FIG 4:**
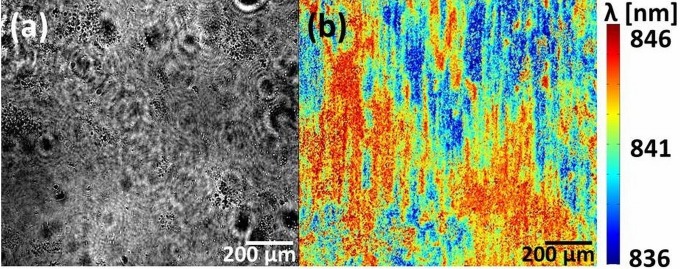
Comparison of the same field of view of a living biofilm on a photonic crystal. (a) Bright-field image; (b) surface resonance image.

While traditional microscopy techniques provide information about cellular morphology and general appearance, surface-enhanced imaging provides information that is specific to the interface between the cell and its substrate without using cytotoxic staining agents or temporally unstable fluorophores. One such surface imaging method is based on photonic crystal resonant surfaces. These structures rely on the excitation of surface resonances, which are very sensitive to changes in the refractive index within ∼200 nm of the surface. Imaging at different wavelengths (“hyperspectral imaging“) then allows one to determine the resonance wavelength at every pixel in the field of view (89). Resonant surfaces therefore offer a unique combination of high sensitivity and spatial information, which makes it possible to map refractive index changes at the very surface of the sensor. Figure 4b is an example of a resonance image of living biofilm obtained after 24 h of incubation, where a much higher image contrast than that of a regular bright-field image (Fig. 4a) of the film is observed.

The spatial resolution of such sensors varies with the refractive index contrast and is in the range of 1.5 to 6 μm (90, 91).

A similar surface resonance technique, called surface plasmon resonance imaging (SPRI), is capable of detecting changes in the refractive index at a metal-dielectric interface. Since SPRI detects a change in particle resonance, the spatial resolution is higher than that for the photonic crystal case. For example, SPRI was used to visualize five different types of cells with lateral resolutions of 0.3 to 0.6 μm, which is thereby close to the diffraction limit (92). Moreover, SPRI can detect the change in the refractive index of an individual mast cell (RBL-2H3) in response to an antigen (IgE, 50 ng/ml) (93, 94). The mechanism of the refractive index changes during living-cell reactions; however, it remains largely unclear. Kosaihira and Ona suggested that the mitochondrial membrane potential generated by the mitochondrial electron-transport chain is related to the refractive index change in living cells (95). Both surface resonance imaging techniques mentioned in this chapter, i.e., photonic crystal and plasmon based, offer a combination of high sensitivity and spatial resolution. The photonic crystal resonances tend to be sharper and hence provide higher sensitivity, while the plasmonic resonances are better confined, thereby providing higher spatial resolution.

## BIOFILM FIELD SENSORS

Analyzing biofilms in natural aquatic environments helps us to improve our understanding of biofilm formation dynamics and our ability to control surface colonization. An example for a nonlaboratory-based sensor was presented by Tamachkiarow, Flemming, and their colleagues (96, 97). The sensor detected the light scattered from particles deposited on the tip of an optical fiber, and it was mounted in a water pipe system. The authors used two multimode fibers, one for illumination and one for detection, with a detection diameter of 200 μm. The intensity of the backscattered light signal was then correlated to a surface bacterial cell density of 10^5^ to 10^10^ cells/cm^2^. While very simple, the drawback of this system is that not only is it selective to microbial deposits, it also incorporates backscattering from abiotic particles and unattached microorganisms of the surrounding bulk water (98). Recently, we developed a biofilm field sensor based on the detection of the natural fluorescence of tryptophan, which is excited by a UV light-emitting diode (LED) at an excitation wavelength (λ_ex_) of 280 nm and is detected at an emission wavelength (λ_em_) of 350 nm. We found a lower detection limit of 4 × 10^3^ cells/cm^2^, corresponding to a surface coverage of 0.01% (33, 99), which is among the lowest limits of any biofilm sensor. With this sensor, we quasi-continuously measured, for the first time, the initial attachment phase and diurnal variations during biofilm formation in the marine environment (100).

Another field sensor system, demonstrated by Strathmann et al., is capable of measuring fluorescence, refraction, transmission, and scattering simultaneously in a water pipe system (101). The sensor uses the intrinsic fluorescence of tryptophan (λ_ex_ = 290 nm and λ_em_ = 340 nm) and, in order to detect metabolic activity, the intrinsic fluorescence of the coenzyme reduced NAD (NADH) (λ_ex_ = 340 nm and λ_em_ = 460 nm). In addition, general deposits and especially inorganic particles were investigated by detection of the backscattered light at a wavelength of 810 nm. Based on the tryptophan fluorescence signal at a λ_em_ of 340 nm, the detection limit for the biomass of this sensor is ∼10^6^ cells/cm^2^, and based on the NADH signal at a λ_em_ of 460 nm, it is ∼10^5^ cells/cm^2^.

## BIOFILM SENSOR SELECTION GUIDELINES AND CONCLUDING REMARKS

A universal optical biofilm sensor, i.e., one that measures all of the interesting parameters, such as thickness, cell density, chemical composition, and formation mechanism, has not yet been reported. Arguably, such a sensor is not required, as a subset of these parameters may be sufficient depending on the line of inquiry that is being pursued. Therefore, the selection of a suitable sensor depends on the following boundary conditions.

## (i) Environment.

- Monitoring of biofilm formation dynamics in the field has to meet challenges that differ from the objectives of highly sophisticated laboratory instrumentation. The crucial issues for biofilm field sensors are their robustness, portability, low power consumption, and minimal assembly of all optical and electronic parts. Depending on the application, a detection range that provides the opportunity to monitor microbial colonization from initial attachment of bacterial cells to an established and mature biofilm under field conditions is required. This process may take days to weeks, calling for an autonomous sensor with a possible sampling frequency in the range of a few minutes to detect short-term variations of microbial settlement. All of the field sensors discussed above fulfill these requirements in terms of robustness, portability, and sampling frequency, yet the detection of the initial attachment phase of bacteria has been shown only by Fischer et al. (100).
- If the environment is highly contaminated with inorganic particles, which may adhere to the surface that is being investigated, then selective detection of the biofilm is required as a means to distinguish between organic and inorganic material on the surface. In this respect, fluorescence- and spectroscopy-based sensors offer advantages, as they directly identify the signature of the organic molecule. Spectroscopy is typically more difficult to perform than, e.g., light scattering, so this added information comes at the disadvantage of added complexity and cost.
- When submersing the entire sensor into an aquatic environment, systems operating in reflection rather than in transmission are much more suitable, as otherwise the light source and the detector will also be overgrown by the microorganisms. Several sensor types do operate in reflection and therefore fulfill this criterion (33, 34, 37, 62, 64, 65, 71, 88, 90, 93, 98, 102, 103).
- Taking into account the fact that the overlaying bulk water often contains organic material, the sensor system should have an effective background signal suppression mechanism or, ideally, should detect only a thin surface sheet, e.g., ATR and photonic crystal resonant surfaces.

## (ii) Expected information.

- In most sensors, the output signal can be correlated with the thickness of the biofilm or cell number; however, this requires microscopy-assisted calibrations of the overall sensor response by reference samples to extract the respective conversion factors and to ensure the linear response of the sensor.
- For sensing microbial activity or exploring the chemical composition and for identifying specific species within the biofilm community, spectroscopic methods, such as fluorescence, FTIR, Raman, and photoacoustic spectroscopy, are most advantageous; however, the complexity and cost of the sensor system will increase.
- If the interface between the cell and the substrate is of interest, then evanescent field sensors and surface resonance imaging are the methods of choice.

## (iii) Sensitivity and selectivity.

- Sensors measuring the thickness of biofilms have been proven to be useful to detect thicknesses ranging from 1 μm to a few millimeters, which make them suitable for aquatic environments where a high number of microorganisms will settle on the surface; however, they are mostly unsuitable for sensing a monolayer of cells.
- To overcome this problem, evanescent field sensors may be appropriate where the penetration depth is limited to hundreds of nanometers, allowing for the probing of a fraction of the microbial cell layer.
- Selectivity is provided by fluorescence, absorption, and Raman scattering sensors, as they are able to excite specific molecules within the biofilm.

Overall, while many different sensor types have now been introduced, we believe that novel sensor concepts are needed to detect biofilms in natural environments, pipe systems, bioreactors, and medical devices, especially at low cost. The future development of biofilm sensors should focus on the miniaturization of the sensor systems using microfabrication technology, which offers the potential for mass production and substantial cost reduction. To grow specific biofilms *in situ* that are hosting human pathogens, such as Vibrio cholerae (104), or to show antimicrobial resistance, the settling substrate of the biofilm sensors can be functionalized with specific antibodies or aptamers (105, 106), which selectively attracts bacteria in the bulk water to attach to the sensor surface.

Moreover, functionalization of the sensor surface allows for exploring microbial community interactions while spotting an array of specific RNA aptamers targeting different bacterial groups. Furthermore, miniaturized biofilm sensors can be integrated in biomedical devices for monitoring dental caries, infections of wounds, gastric ulcers, or catheters.
